# Minimum 5-Year Outcomes of Dorsal Intercarpal Ligament Capsulodesis With Scapholunate Interosseous Ligament Repair for Subacute and Chronic Static Scapholunate Instability: A Clinical Series of 5 Patients

**DOI:** 10.1016/j.jhsg.2022.01.007

**Published:** 2022-02-17

**Authors:** Hiroki Shibayama, Yuichiro Matsui, Daisuke Kawamura, Daisuke Momma, Takeshi Endo, Norimasa Iwasaki

**Affiliations:** ∗ Department of Orthopaedic Surgery, Faculty of Medicine and Graduate School of Medicine, Hokkaido University, Sapporo, Hokkaido, Japan; † Center for Sports Medicine, Hokkaido University Hospital, Sapporo, Hokkaido, Japan

**Keywords:** Dorsal intercarpal ligament capsulodesis, Long-term outcomes, Scapholunate interosseous ligament repair, Subacute and chronic static scapholunate instability

## Abstract

**Purpose:**

Treatment of subacute and chronic static scapholunate instability remains challenging. We aimed to determine 5- to 10-year outcomes of dorsal intercarpal ligament capsulodesis with scapholunate interosseous ligament repair for subacute and chronic static scapholunate instability.

**Methods:**

Six patients with subacute and chronic static scapholunate instability underwent dorsal intercarpal ligament capsulodesis with scapholunate interosseous ligament repair between 2011 and 2015, and 5 of them were followed for at least 5 years after surgery. The clinical and radiological results were retrospectively investigated. All patients were male, and the mean age at surgery was 37 years (range, 21–47 years). The mean period from injury to surgery was 26.2 months (range, 2–113 months). The surgical procedure was a modification of a method reported by Szabo et al.

**Results:**

The mean postoperative follow-up period was 8.1 years (range, 5.1–9.5 years). Median Disabilities of the Arm, Shoulder, and Hand and Mayo wrist scores improved from 23.3 to 1.7 and from 55 to 80, respectively, from before surgery to the final follow-up. Although the median flexion angle tended to be smaller, the median extension angle tended to be greater than before surgery. The median percent grip strength increased from 72.3% before surgery to 99.2% at the final follow-up. The median scapholunate gap improved from 4.2 mm before surgery to 2.1 mm at the final follow-up. The median scapholunate angle also improved from 95.7° before surgery to 71.3° at the final follow-up. Osteoarthritic changes were observed in 2 of 5 patients at the final follow-up.

**Conclusions:**

The scapholunate gap in all patients was within the normal range after a mean of 8.1 years of follow-up. Dorsal intercarpal ligament capsulodesis with scapholunate interosseous ligament repair is considered a good alternative for subacute and chronic static scapholunate instability based on these 5- to 10-year outcomes.

**Type of study/level of evidence:**

Therapeutic IV.

Scapholunate instability (SLI) is the most common form of carpal instability. It causes wrist dysfunction, negatively affects quality of life, and, if left untreated, can lead to scapholunate advanced collapse wrist.^1^^,^^2^ Many treatment methods for SLI, such as tenodesis, capsulodesis, bone-tissue-bone autograft, and their combinations, have been reported.^3^, ^4^, ^5^, ^6^, ^7^, ^8^, ^9^, ^10^, ^11^ In recent years, new arthroscopic procedures, ligamentoplasty, and the scapholunate axis method have been reported.^12^, ^13^, ^14^ Scapho-trapezio-trapezoid arthrodesis has not been reported in recent years because of a series of reports describing poor long-term outcomes.^15^^,^^16^ According to some systematic reviews, there were no significant differences in outcomes among the treatments.^17^^,^^18^ However, it was pointed out that many of the reports had low evidence levels and that long-term comparative studies were required.^19^^,^^20^

Scapholunate instability can be classified as acute, subacute, or chronic and as dynamic or static.^21^^,^^22^ In general, acute SLI denotes development within 3 weeks after the injury, subacute denotes development more than 3 weeks and less than 12 weeks after the injury, and chronic denotes development more than 12 weeks after the injury.^23^^,^^24^ Static SLI denotes that the scapholunate gap (SLG) exceeds 3 mm on posteroanterior radiographs of the wrist at rest. In contrast, dynamic SLI denotes that the SLG is normal on posteroanterior radiographs at rest, but exceeds 3 mm on stress radiographs with a power grip. Chronic static SLI is considered more severe and tends to have poorer outcomes than acute dynamic SLI, even after surgical treatment. Although the traditional type of capsulodesis is considered to be indicated for dynamic SLI, according to some reports it does not maintain the carpal alignment over time in patients with chronic static SLI.^7^, ^8^, ^9^^,^^23^, ^24^, ^25^

There are various methods for capsulodesis. Among these, Szabo et al^25^^,^^26^ reported dorsal intercarpal ligament (DICL) capsulodesis with scapholunate interosseous ligament (SLIL) repair. We believe that firm repair of the SLIL is the most important factor in the treatment of SLI, and perform operations with some modifications of the Szabo procedure. We hypothesized that good 5- to 10-year outcomes can be achieved by DICL capsulodesis with SLIL repair for subacute and chronic static SLI. The aim of this observational study was to evaluate the minimum 5-year outcomes of this procedure for subacute and chronic static SLI.

## Materials and Methods

### Patients

Six patients with subacute and chronic static SLI underwent surgery between 2011 and 2015, and 5 of them were followed for at least 5 years after surgery. The main surgical indications were wrist pain and radiographic findings. The operations were performed by 2 hand surgery specialists (N.I. and Y.M.). These 5 cases were retrospectively investigated. All patients were male, and the mean age at surgery was 37 years (range, 21–47 years). The mean period from injury to surgery was 26.2 months (range, 2–113 months) (Table 1). The preoperative SLG exceeded 3 mm in all patients, and all cases were classified as having subacute and chronic static SLI. This was a retrospective case review that adhered to the tenets of the Declaration of Helsinki and was approved by the institutional review board of Hokkaido University (020-0083), and written informed consent was obtained from all patients.

**Table 1:** Patient Demographic Data

Patient	Age, y	Affected Hand	Dominant Hand	Follow-Up Time, mo	Time From Injury, mo

1	21	right	right	114	8
2	47	left	right	114	4
3	44	right	right	109	113
4	34	left	right	87	2
5	39	left	right	62	4
Mean	37			97.2	26.2

### Surgical technique

**Figure 1:**
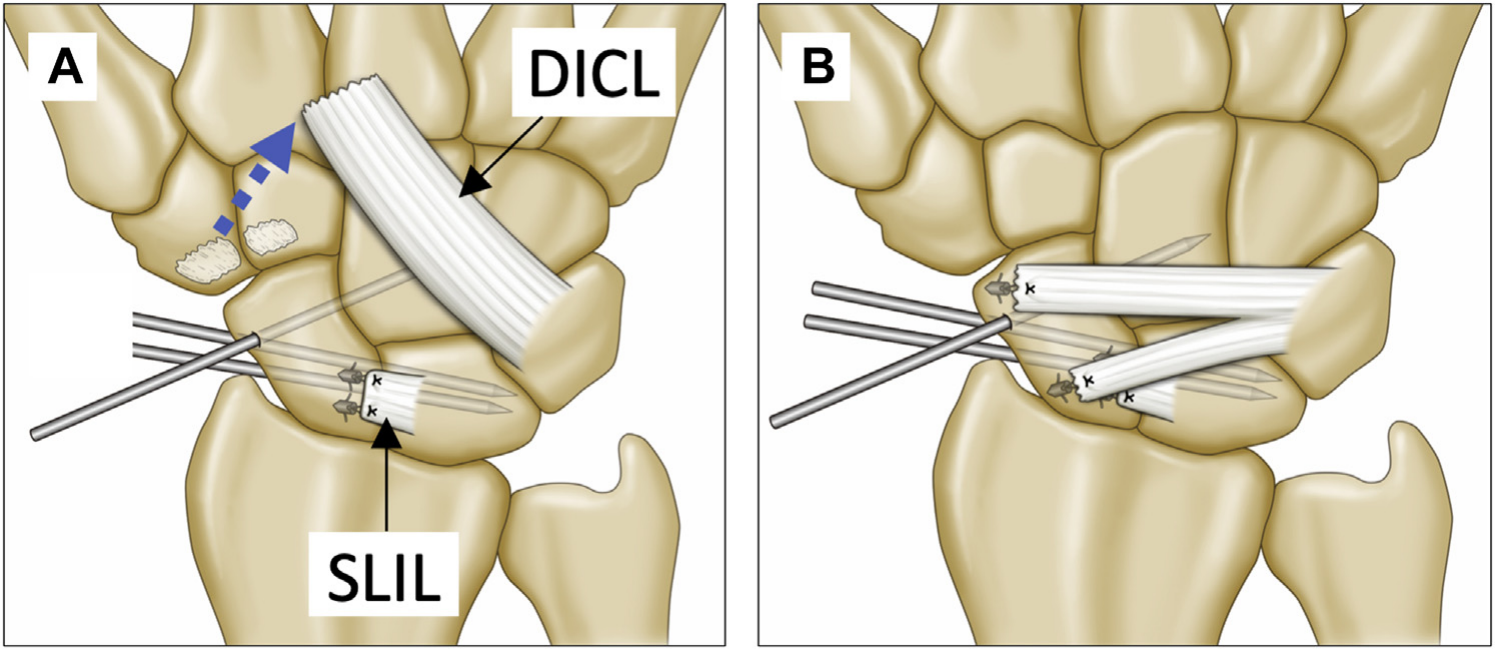
Schematic representation of our modification. **A** After dissection of the DICL and K-wire fixation, the SLIL was firmly repaired. The blue dotted arrow indicates that the DICL was lifted off of its origin. **B** The DICL was bifurcated and both parts were sutured to the scaphoid.

Surgery was conducted according to a modification of the procedure reported by Szabo.^26^ Employing a dorsal approach to the wrist, the DICL was dissected and raised based on the ulnar side (Fig. 1A). The SLIL was avulsed from the scaphoid in all cases. The scaphoid and lunate were anatomically reduced using the joystick method and were fixed with 2 K-wires. The scaphoid and the capitate were also fixed with a K-wire, and carpal alignment was confirmed using fluoroscopy. The most important procedure was repairing the remnants of the SLIL carefully and firmly using 1 or 2 suture anchors with bone decortication to maintain a normal SLG (Mitek mini 1.8 mm, 2-0 nonabsorbable suture [Depuy Mitek] or JuggerKnot mini 1.0 mm, 2-0 or 3-0 nonabsorbable suture [Biomet Sports Medicine]) (Fig. 1A). The DICL was then split in half, in line with its fibers, while maintaining the proximal attachment site. These fibers were then reattached under as much tension as possible to the distal and proximal scaphoid using the same 2 types of suture anchor (Fig. 1B). The distal fibers of the bifurcated DICL were used to maintain the scapholunate angle (SLA) and the proximal fibers were used to maintain the SLG. After irrigation, the dorsal capsule was sutured and skin closure was performed. A long-arm cast was applied for postoperative immobilization. The K-wire between the scaphoid and capitate was removed 6 weeks after surgery in an outpatient office, and the cast was removed at the same time. The K-wires between the scaphoid and lunate were buried under the skin and removed 8–12 weeks after surgery in an operating room.

### Outcome measures

The clinical results were measured using the Disabilities of the Arm, Shoulder, and Hand (DASH) score, Mayo wrist score, range of motion of the wrist, and grip strength before surgery and at the final follow-up. Grip strength was measured using a Smedley dynamometer (Hand Dynamometer, MIS). Grip strength is presented as the percentage obtained by dividing the grip strength of the affected side by that of the contralateral side and multiplying by 100. The radiographic results were evaluated by measurement of the SLG and SLA before surgery and at the final follow-up. An investigator (H.S.) who had completed equivalent training as a hand surgery specialist but was not involved in performing the surgery evaluated the radiographic findings. Osteoarthritic changes were examined at the final follow-up. The SLG was defined as the distance between the scaphoid and lunate at the midpoint of Gilula’s lines on a posteroanterior radiograph taken at rest with the shoulder adducted, the elbow flexed, and the forearm in a neutral position. The SLA was defined as the angle between a tangential line connecting the 2 palmar convexities of the scaphoid and a perpendicular line connecting the distal articular surfaces of the lunate on a lateral radiograph of the wrist.

## Results

**Figure 2:**
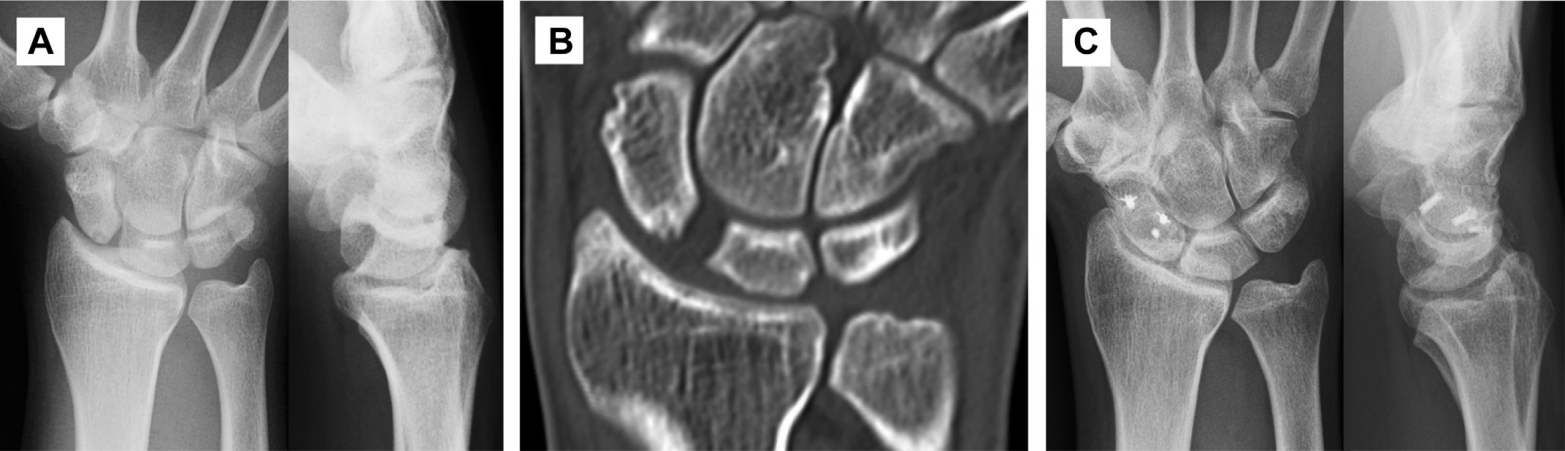
Radiological findings of patient 1. **A** and **B** Preoperative radiographs and computed tomographic imaging showing scapholunate instability (SLG: 4.2 mm; SLA: 97.2°). **C** Radiographs at 9.5 years after surgery showing normal carpal alignment (SLG: 1.6 mm;SLA: 60.7°). Osteoarthritic changes such as a slight narrowing of the joint space and sclerosis of the subchondral bone at the radioscaphoid joint were observed.

The mean postoperative follow-up period was 8.1 years (range, 5.1–9.5 years). Because the SLIL was totally ruptured and the carpal alignment was easily reducible in all cases, we diagnosed the cases as having stage 4 instability by the Garcia-Elias classification.^6^ The median DASH score and median Mayo wrist score were improved from 23.3 to 1.7 and from 55 to 80, respectively, from before surgery to the final follow-up (Table 2). The patients with the lower DASH scores had pain at work and during sports activities. Although the median flexion angle tended to be smaller, the median extension angle tended to be greater. The median percent grip strength increased from 72.3% before surgery to 99.2% at the final follow-up. According to radiological findings, the median SLG improved from 4.2 mm before surgery to 2.1 mm at the final follow-up. The median SLA also improved from 95.7° before surgery to 71.3° at the final follow-up. Osteoarthritic changes, comprising a slight narrowing of the joint space and sclerosis of the subchondral bone at the radioscaphoid joint (Fig. 2; Fig. E1, available online on the *Journal*’s website at www.jhandsurg.org), were observed at the final follow-up in 2 cases. These cases were not symptomatic. Their DASH scores were improved from 16.7 to 1.7 and from 43.3 to 7.5. Mayo wrist scores were also improved, from 65 to 90 and from 45 to 80.

**Table 2:** Clinical and Radiological Outcomes of 5 Patients

Patient	DASH		MWS		Extension, °		Flexion, °		Grip Strength, %		SLG, mm		SL Angle, °	
	Before surgery	Final follow-up	Before surgery	Final follow-up	Before surgery	Final follow-up	Before surgery	Final follow-up	Before surgery	Final follow-up	Before surgery	Final follow-up	Before surgery	Final follow-up
1	16.7	1.7	65	90	80	80	80	54	91.1	95.8	4.2	1.6	97.2	60.7
2	3.3	2.5	75	85	80	70	80	65	93.5	95.8	4.9	2.3	92.5	73.1
3	43.3	7.5	45	80	70	70	60	45	103.3	124.1	4.5	2.8	94.8	86.1
4	23.3	0	35	80	30	80	50	65	13	88.1	4.5	1.8	103.3	65.6
5	32.1	0	55	80	80	80	70	35	60.5	92	3	2	90.8	71
Median	23.3	1.7	55	80	80	80	70	54	91.1	95.8	4.5	2	95	71
IQR	16.7–32.1	0–2.5	45–65	80–85	70–80	70–80	60–80	45–65	60.5–93.5	92–95.8	4.2–4.5	1.8–2.3	92.5–97.2	65.6–73.1

IQR, interquartile range; MWS, Mayo wrist score.

## Discussion

Some authors reported that capsulodesis tends to provide poor outcomes for subacute and chronic static SLI, and there are few reports of long-term follow-ups.^7^, ^8^, ^9^^,^^25^^,^^27^^,^^28^ Therefore, we aimed to determine 5- to 10-year outcomes of DICL capsulodesis with SLIL repair for subacute and chronic static SLI. Our experience with this procedure is encouraging. The 5- to 10-year outcomes in our case series demonstrate substantial clinical and radiological improvements.

Many reports have stated that capsulodesis is a good indication for dynamic SLI, reflecting mild cases of SLI, but it is not recommended for subacute and chronic static SLI.^17^^,^^25^ Gajendran et al^25^ reported long-term outcomes of DICL capsulodesis with SLIL repair in 15 wrists with subacute and chronic static SLI. Although the clinical symptoms and wrist functions were generally improved, they found it difficult to maintain the radiographic alignment after a mean of 86 months of follow-up. The mean preoperative SLG (4 mm) was unchanged at the final follow-up, and osteoarthritis occurred in 8 cases (53.3%). However, in the present study, SLG was maintained at less than 3 mm in all cases, even after 5–10 years of follow-up. This difference may be related to our modifications: in particular, the use of 2 suture anchors for SLIL repair and reinforcement of the SLIL with the bifurcated DICL. Reduction of the SLG—that is, refinement of the dissociation between the scaphoid and lunate—is considered the most important factor contributing to better outcomes of SLI treatment. Even in cases where the injury occurred a long time ago, there may still be remnants of the SLIL to enable repair. However, in the case of patient 3, the interval between the injury and the operation was more than 10 years. The SLG was 2.8 mm at the final follow-up, and this is the maximum value of the 5 cases of this study despite this patient having undergone the same procedures as the others. In chronic cases, the intercarpal ligaments may also be attenuated. The remnants should be dissected carefully and repaired firmly with 1 or 2 suture anchors. Surgical techniques to reinforce the repaired SLIL should also be performed using bifurcation of the DICL.

We believe that the results of the present study are extremely useful. Although new surgical procedures are still being developed for SLI, our procedure is less invasive and simpler than other surgical procedures. Recently, there has been a trend toward ligament reconstruction on both the dorsal and volar sides of the wrist; however, our procedure can be performed on the dorsal side only.^3^^,^^12^ In addition, for tenodesis and ligamentoplasty, it is necessary to make a bone tunnel, and this is associated with a risk of carpal bone necrosis and scaphoid-trapezio-trapezoid osteoarthritis.^29^, ^30^, ^31^ For tenodesis, the flexor carpi radialis and the extensor carpi radialis longus, which are considered the volar and dorsal stabilizers of the scaphoid, are often partially dissected, and disadvantages of their sacrifice have been reported.^32^ Compared with bone-tissue-bone autografts and ligamentoplasty using the palmaris longus tendon, our procedure does not require the sacrifice of a healthy tendon or bone and can be considered a minimally invasive procedure. Although arthroscopic procedures are less invasive, they have some disadvantages, such as the procedures’ complexity, technically demanding nature, and steep learning curve. The postoperative flexion angle tends to decrease because our procedure is a type of dorsal capsulodesis. Indeed, the flexion angle had decreased at the final follow-up in 4 patients. This technique improves carpal kinematics, with an example being improvement in the SLGs; however, there is still some residual scaphoid rotation or carpal pronation. Minor osteoarthritic changes were found in 2 patients, both of whom had little pain.

The limitations of the present study are the retrospective nature, small number of cases, and lack of a control group. We believe that the advantages of our surgical procedure will become clear in the future with the accumulation of long-term follow-up data for more cases. Regarding the presence of osteoarthritis, it is necessary to continue follow-up for these cases for a long period of time.

The 5- to 10-year outcomes of DICL capsulodesis with SLIL repair were acceptable for subacute and chronic static SLI. Although these results are encouraging, a larger series with similar follow-ups and multiple surgeons would be beneficial in further clarifying the outcomes of this procedure.

## Supplementary Data

Figure E1 Preoperative and final follow-up radiographs of the 4 cases, other than the case in Figure 2.
