# Supplementary material for: Minimum 5-Year Outcomes of Dorsal Intercarpal Ligament Capsulodesis With Scapholunate Interosseous Ligament Repair for Subacute and Chronic Static Scapholunate Instability: A Clinical Series of 5 Patients
Source: J Hand Surg Glob Online. 2022 Feb 17;4(3):162–5. doi: 10.1016/j.jhsg.2022.01.007 (PMC9120793; doi:10.1016/j.jhsg.2022.01.007)
Supplement: Figure E1 — Preoperative and final follow-up radiographs of the 4 cases, other than the case in Figure 2. [file mmc1.pptx]

## Slide 1
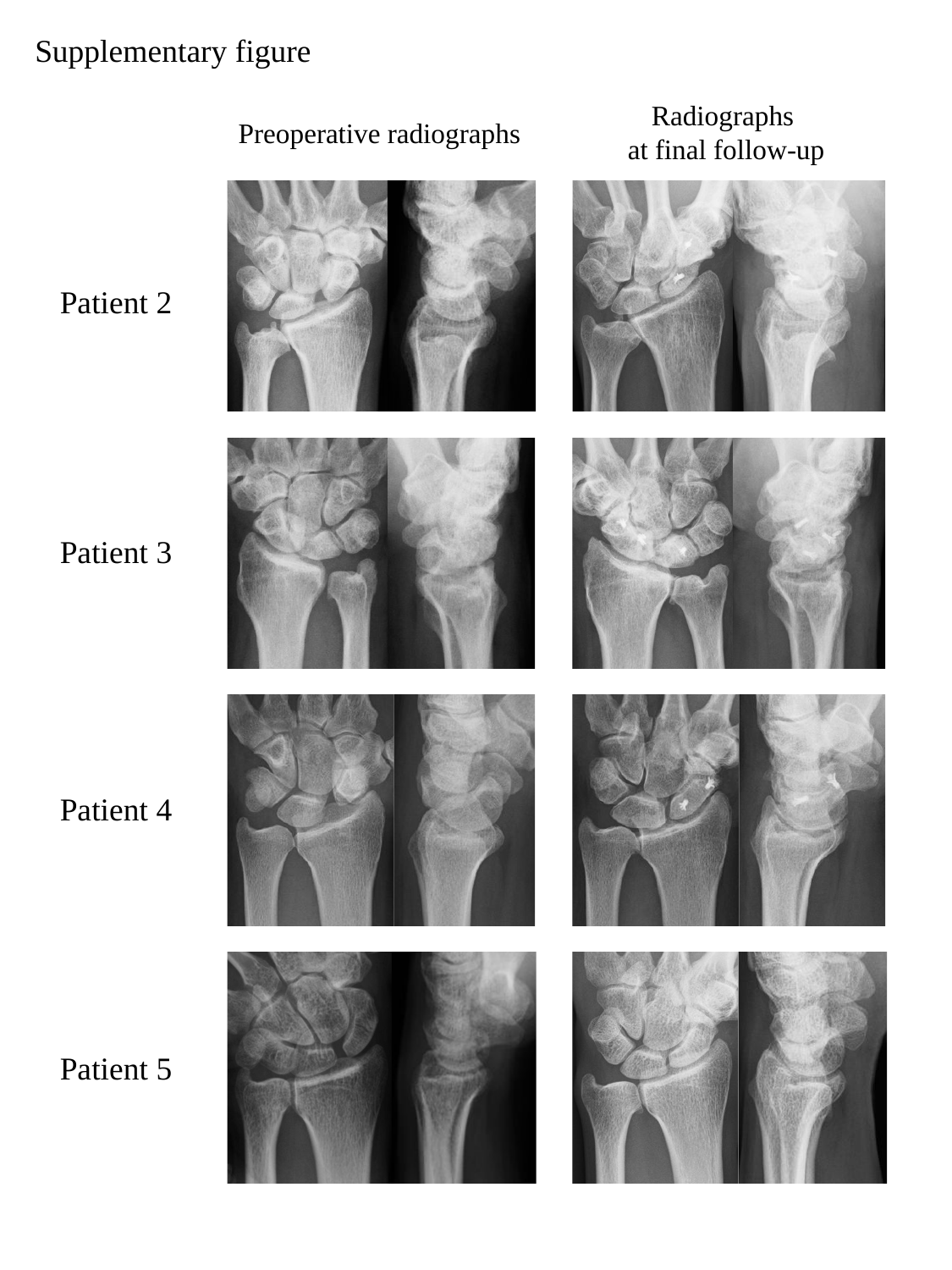

Supplementary figure
Radiographs
 at final follow-up
Preoperative radiographs
Patient 2
Patient 3
Patient 4
Patient 5
